# Distinct prognostic value of circulating anti-telomerase CD4^+^ Th1 immunity and exhausted PD-1^+^/TIM-3^+^ T cells in lung cancer

**DOI:** 10.1038/s41416-019-0531-5

**Published:** 2019-07-30

**Authors:** Caroline Laheurte, Magalie Dosset, Dewi Vernerey, Laura Boullerot, Béatrice Gaugler, Eléonore Gravelin, Vincent Kaulek, Marion Jacquin, Laurie Cuche, Guillaume Eberst, Pascale Jacoulet, Elizabeth Fabre, Françoise Le Pimpec-Barthes, Eric Tartour, Marcelo De Carvalho Bittencourt, Virginie Westeel, Olivier Adotévi

**Affiliations:** 1 University Bourgogne Franche-Comté, INSERM, EFS BFC, UMR1098, Interactions Hôte-Greffon-Tumeur/Ingénierie Cellulaire et Génique, F-25000 Besançon, France; 2 INSERM CIC-1431, Clinical Investigation Center in Biotherapy, Plateforme de Biomonitoring, F-25000 Besançon, France; 3 Department of medical Oncology, Methodology and Quality of Life Unit in Oncology, University Hospital of Besançon, F-25000 Besançon, France; 4 UPMC Univ Paris 06, INSERM UMR 938, Centre de Recherche Saint-Antoine, Sorbonne Universités, F-75012 Paris, France; 5 Department of Pneumology, University Hospital of Besançon, F-25000 Besançon, France; 6 Department of medical Oncology, Assistance Publique-Hôpitaux de Paris, Hôpital Européen Georges Pompidou, Paris, France; 7 Department of Thoracic surgery, Assistance Publique-Hôpitaux de Paris, Hôpital Européen Georges Pompidou, Paris, France; 8 Department of Biological Immuology, Assistance Publique-Hôpitaux de Paris, Hôpital Européen Georges Pompidou, Paris, France; 9 University Hospital of Nancy and CECITA team, IMOPA UMR7365 University of Lorraine/CNRS, Laboratory of Immunology, Vandoeuvre-les-Nancy, France

**Keywords:** Translational immunology, Non-small-cell lung cancer, Prognostic markers

## Abstract

**Background:**

Despite the critical roles of Th1-polarised CD4^+^ T cells in cancer immunosurveillance, the translation of their potential to clinical use remains challenging. Here, we investigate the clinical relevance of circulating antitumor Th1 immunity in non-small cell lung cancer (NSCLC).

**Methods:**

The circulating antitumor Th1 response was assessed by the ELISpot assay in 170 NSCLC patients using a mixture of HLA class II-restricted peptides from telomerase (TERT). Phenotyping of blood immune cells was performed by flow cytometry.

**Results:**

TERT-reactive CD4 T-cell response was detected in 35% of NSCLC patients before any treatment. Functional analysis showed that these cells were effector memory and Th1 polarised capable to produce effector cytokines, such as IFN-γ, TNF-α and IL-2. The presence of anti-TERT Th1 response was inversely correlated with the level of exhausted PD-1^+^/TIM-3^+^CD4 T cells. The level of these two immune parameters differentially affected the survival, so that increased level of anti-TERT Th1 response and low rate of exhausted PD-1^+^TIM-3^+^CD4^+^ T cells were associated with a better prognosis.

**Conclusions:**

Systemic anti-TERT Th1 response plays a strong antitumor protective role in NSCLC. This study underlines the potential interest of monitoring circulating antitumor Th1 response for patients’ stratification and therapy decision.

## Background

A functional adaptive immune system, in which tumour antigens are recognised as foreign and eliminated, is fundamental for preventing cancer development and progression.^1^ Research on cancer immunity and immunotherapy has mainly focused on the antitumor activity of cytotoxic CD8^+^ T cells (CTL), but cumulative data also highlight a major role of CD4^+^ T cells. Among the subpopulations of CD4^+^ helper T cells, CD4^+^Th1 subset that produces IFN-γ, TNF-α and interleukin-2 play a well-defined role in antitumor protection by orchestrating cell-mediated immunity against cancer cells.^2,3^ This cell subset shows the capacity to enhance tumour-specific CD8^+^ T-cell generation, function and memory.^4–6^ Tumour-reactive CD4^+^ Th1 T cells also promote the secretion of chemo attractants that support the entry of effector cells into the tumour site.^7,8^ Emerging properties of CD4^+^ helper T cells also indicate their involvement in inhibiting tumour angiogenesis,^9^ promoting cancer cell senescence,^10^ highly sensitive “neoepitope” recognition^11,12^ and protecting effector CTLs from exhaustion.^6,8^ In many human cancers, a Th1-polarised CD4^+^ T-cell signature within the tumour microenvironment (TME) is associated with better survival.^13,14^ Furthermore, evidence indicates that targeting CD4^+^ T cells can significantly increase cancer immunotherapy efficacy, and may induce more durable tumour control than targeting CD8^+^ T cells.^15,16^ These properties underscore the importance of finding ways to better harness the clinical potential of CD4^+^ helper T cells.

The comprehensive monitoring of tumour-reactive CD4^+^ T cells is hampered by several hurdles, such as tumour antigen selection, HLA class II polymorphism, low frequencies of antigen-specific CD4^+^ T cells and the plasticity of CD4^+^ helper T cells.^17^ One approach to circumventing these obstacles involves the ability of CD4^+^ T cells to recognise degenerate HLA class II-restricted epitopes from relevant shared tumour-associated antigens.^18–21^

In this study, we investigated the clinical significance of circulating antitumor CD4^+^ Th1 response in patients with non-small cell lung cancer (NSCLC). To this end, we quantified functional telomerase (TERT)-reactive CD4 T cells as a surrogate marker of antitumor Th1 response by using a mixture of HLA class II-restricted peptides.^22–24^ Our results showed an unexpected relationship between the circulating TERT-reactive CD4^+^ Th1 response and accumulated exhausted PD-1^+^/TIM-3^+^ CD4^+^ T cells. It appears that a robust circulating anti-telomerase CD4^+^ Th1 response plays a strong protective role in NSCLC patients, while high level of exhausted PD-1^+^/TIM-3^+^ CD4 T cells in peripheral blood is associated with poor prognosis. Thus, the level of adaptive antitumor CD4^+^ Th1 immunity in peripheral blood could be used for NSCLC stratification.

## Methods

### Patients and study design

The TeloCap01 study is a prospective multicentric immunomonitoring study conducted in patients with stage I–IV NSCLC. The primary objective of this study was to evaluate the landscape of telomerase-specific CD4^+^ T-cell responses in patients with NSCLC. Between July 2010 and January 2014, 170 patients with NSCLC were included from the University Hospital of Besançon (Besançon) and the European Hospital Georges Pompidou (Paris). Before any therapy, including surgery, we collected and isolated blood lymphocytes, sera and plasma, which were frozen until use. Survival data were collected at 1 and 2 years after inclusion. Blood cells were also collected from anonymous healthy donors from the Etablissement Français du Sang (EFS, Besançon, France), following EFS guidelines. All patients and healthy donors gave their signed informed consent, and the protocol was approved by local ethic committees and the French national drug agency (N°EUDRACT: 2009-A00642-55).

### Synthetic tumour antigen-derived peptides

To measure telomerase-specific CD4^+^ T-cell responses in blood, we used a mixture of eight highly promiscuous HLA-DR and HLA-DP4-binding 15-mer peptides derived from telomerase (TERT), which has been previously described by our group.^24–26^ In some experiments, we used mixtures of 15-mer peptides derived from NY-ESO-1 or Wilms tumour (WT-1). To evaluate the antiviral T-cell responses, we used peptide mixtures derived from influenza virus (Flu), Epstein Barr virus (EBV) and cytomegalovirus (CMV) (PA-CEF-001), which were purchased from JPT (Germany) or CTL (Cellular Technology Ltd, Germany) at >80% purity.

### In vitro stimulation for the detection of tumour-reactive CD4^+^ Th1 responses in blood

Telomerase-specific CD4^+^ Th1 responses were assessed in peripheral blood mononuclear cells using a standard IFN-γ ELISpot assay, following in vitro stimulation, as previously described.^24–26^ Briefly, PBMCs (3–4 × 10^6^) were cultured for 6 days in 24-well plates in the RPMI with 5% human serum and 1% penicillin–streptomycin, along with the mixture of TERT-derived peptides (5 µg/mL). Recombinant interleukin 7 (IL-7; 5 ng/mL; Peprotech) was added on day 1, and recombinant interleukin-2 (IL-2; 20 UI/mL; Novartis) was added on day 3. In some patients’ samples, stimulation was performed with a mixture of peptides derived from NY-ESO-1 and WT-1. To assess antiviral T-cell responses, cells were stimulated with a mixture of peptides derived from CMV, EBV and Flu (1 µg/mL), following a similar procedure. Then, the presence of specific T cells was measured using IFN-γ ELISpot assay or cytokine intracellular staining.

### IFN-γ ELISpot assay

ELISpot assay was performed according to the manufacturer’s instructions (Diaclone, France). Briefly, lymphocytes from in vitro stimulation (10^5^ per well) were incubated for 18 h at 37 °C in an ELISpot plate pre-coated with anti-human IFN-γ monoclonal antibody, with or without peptide mixtures in the X-vivo 15 medium (Ozyme, BE04-418). Cells cultured with medium alone or PMA/ionomycin (5 µg/mL; Sigma-Aldrich, L2759) were used as negative and positive controls, respectively. IFN-γ-secreting T cells, i.e., spot-forming cells in this assay, were counted using the C.T.L. Immunospot system. After subtracting the negative control values (background), we calculated the number of IFN-γ spots per 10^5^ cells. A response was considered positive if the number of IFN-γ spots per 10^5^ cells was both >10 and more than two times the background.^27^ The results are presented as a ratio, calculated as follows: [(number of spots in the TERT-peptide conditions)−(number of spots in the medium conditions)]/(number of spots in the medium conditions). All experiments were conducted in triplicate.

### Flow cytometry

Absolute numbers of T cells, B cells and NK cells were determined in fresh samples using a single-platform flow cytometry approach, applying the TetraCXP method with Flow-Count fluorospheres (Beckman Coulter, Villepinte, France) and TetraCHROME antibodies (CD45/CD4/CD8/CD3/CD19/CD56, Beckman Coulter), according to the manufacturer’s recommendations. Blood immune cells, including T_regs_ (CD3^+^CD4^+^CD127^−^CD25^+^FOX-P3^+^) and exhausted T cells (PD-1^+^TIM-3^+^), were measured in thawed PBMCs using flow cytometry. Briefly, PBMCs were thawed and incubated for 10 min at 4 °C with Fixable viability dye (eBioscience), and then for 30 min at 4 °C with the corresponding surface antibodies. In some experiments, Ki-67 staining (Miltenyi) was performed. For intracellular staining (FOXP-3, Ki-67), cells were fixed and permeabilised using the Foxp3/Transcription Factor Staining Buffer Set (00-5523-00, eBioscience), following the manufacturer’s protocol.

For intracellular cytokine staining (ICS), cells were incubated with TERT peptides (5 μg/ml) in the X-vivo 15 medium. After 6 h, BDGolgiPlug™ (BD Biosciences) was added, and cells were stimulated 15 h before staining with antibodies against CD3, CD4, CD8, IFN-γ, TNF- α, IL-2 and IL-17 using DuraClone^TM^ IF T activation or DuraClone^TM^ IF T helper (Beckman Coulter). In some ICS experiments, following markers ICOS, CCR7, CD45RA, CXCR3 and CCR6 were used for phenotypic analysis. Cells were acquired on FACSCanto™ II cytometer (BD Biosciences), and data were analysed using FACSDiva™ and Kaluza™ softwares. A table in the Supplementary Methods presents the complete list of monoclonal antibodies used for immune cells characterisation.

### In vitro blockade of immune checkpoint receptors

PBMC (2 × 10^6^) were cultured in 24-well plates with TERT-derived class II peptides as above and with the following blocking antibodies: anti-PD-1 (Nivolumab, BMS, Pharmacy unit, University Hospital Besançon), anti-TIM-3 (clone F38-2E2, eBioscience) and anti-PD-L1 (clone MIH1, eBioscience). Blocking antibodies (5 µg/ml) were added in the culture at day 0 and day 3. Cells cultured in presence of mouse IgG1 κ (clone P3.6.2.8.1, eBioscience) and human IgG4 (clone ET904, Biolegend) isotypes were used as control for anti-PD-L1/anti-TIM-3 and anti-PD-1 antibodies, respectively. Specific CD4 T-cell responses were measured after 6 days of in vitro stimulation with IFN-γ-ELISpot and ICS.

### Blood cytokines measurement

In the patients’ sera samples, we assessed a panel of cytokines, including IL1β, IL-5, IL6, Il-8, IL-13, IL17, IL10, TNF-α and TGFβ, using a Cytometric Bead Array kit, CBA assay (BD Biosciences), following the manufacturer’s instructions.

### Statistics

Descriptive statistics are described as mean or median, with the interquartile range for continuous variables. The non-parametric Student’s *t* test (Mann–Whitney U-test) was used for two-group comparisons. Categorical variables were expressed as frequency (percentage). Proportions were compared using the χ^2^ test or Fisher’s exact test, as appropriate. We performed hierarchical cluster analysis and constructed dendrograms using the online Morpheus software and robust Z-score normalisation (https://software.broadinstitute.org/morpheus/). To explore the relationship between anti-TERT CD4 Th1 response and all blood immune parameters, we used a principal component analysis (PCA) approach, using the dudi.pca module of the ade4 package of R software (version 2.10.1). For survival analysis according to anti-TERT Th1 response, we determined a threshold using the median ratio (3.7, IQR: 2.6–7.5) of IFN-γ spots between the TERT-derived peptides stimulation and the negative control. Overall survival (OS) was calculated from the date of study enrolment to the date of death from any cause. Surviving patients were censored at the time of their last follow-up assessment. OS was estimated using the Kaplan–Meier method, described using median or rate at specific time points and 95% confidence interval (95% CI), and compared among the groups using the log-rank test. For comparisons among multiple groups, we performed analysis of variance (ANOVA) with Bonferroni correction. Cox proportional hazard models were used to estimate the hazard ratio (HR) and 95% CI for factors associated with OS. We first performed univariate Cox analysis to assess the association of parameters with OS. Then parameters with *P-*values of <0.05 were entered into the final multivariable Cox regression model. To check the assumption of proportionality, we plotted log-minus–log-survival curves and constructed cumulative martingale process plots. All analyses were performed using SAS version 9.4 (SAS Institute, Cary NC), R software version 2.15.2 (R Development Core Team, Vienna, Austria; http://www.r-project) and Prism software version 6 (Graph Pad software, La Jolla, CA, USA). Considering the descriptive and exploratory approaches used, *P-*values were uncorrected for multiple testing. All tests were two sided, and differences were considered statistically significant at the level of *P* *<* 0.05.

## Results

### Circulating TERT-reactive CD4^+^ T cells are effector memory and Th1 polarised in NSCLC patients

**Fig. 1:**
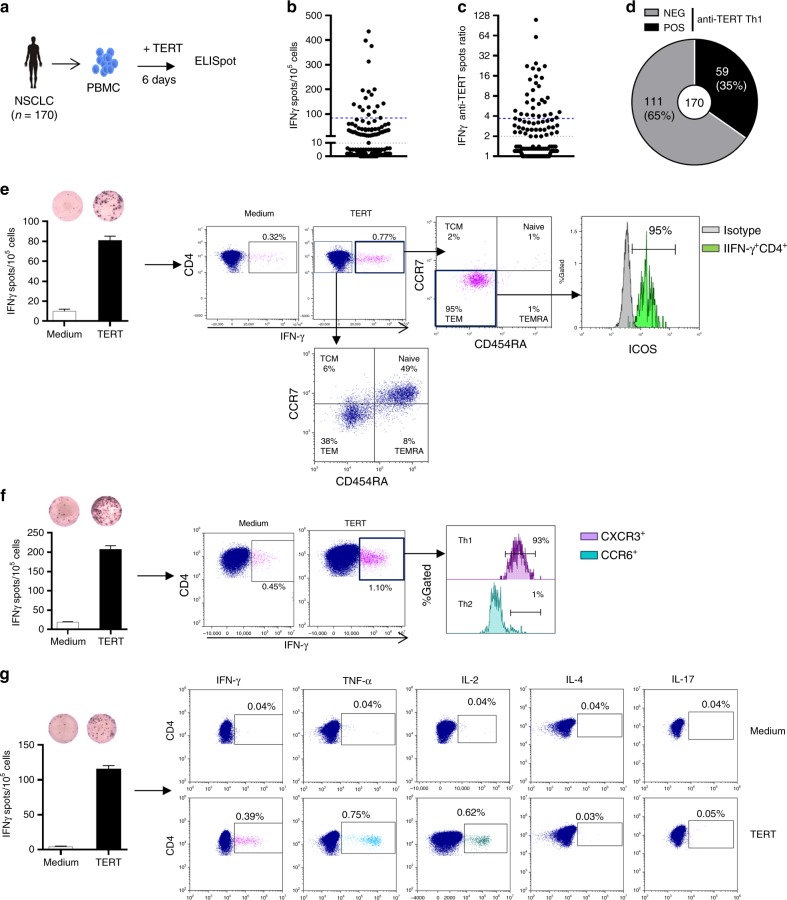
Distribution and functional characterisation of TERT-specific CD4^+^ T cells in patients with NSCLC. **a** TERT-specific CD4^+^ T-cell responses were evaluated in 170 naïve-NSCLC patients by IFN-γ ELISpot assay performed after an in vitro stimulation of PBMC with HLA class II peptides derived from TERT. **b**, **c** Distribution of anti-TERT IFN-γ CD4^+^ T cells in NSCLC patients (*n* = 170), shown as the number of spots (**b**), and ratio of spots (**c**). Grey lines indicate the positivity thresholds, and blue lines indicate the median of spots calculated in responders patients. **d** Frequency of patients with negative (NEG) and positive (POS) anti-TERT Th1 responses. **e**–**g** Phenotypic and functional characterisation of anti-TERT CD4^+^ T cells detected by flow cytometry. **e** Dot plots of one representative patient show CCR7 and CD45RA and ICOS staining in IFN-γ^−^/IFN-γ^+^ CD4^+^ T cells. **f** Dot plots of one representative patient show CXCR3 and CCR6 staining in IFN-γ^+^ CD4^+^ T cells. **g** Dot plots of one representative patient show IFN-γ, TNF-α, IL-2, IL-4 and IL-17 cytokines production in response to TERT stimulation. The data are representative of three independent experiments

To measure pre-existing antitumor CD4^+^ Th1 response in NSCLC patients, we quantified telomerase-reactive CD4^+^ T-cell response by IFN-γ ELISpot assay using a mixture of highly promiscuous HLA class II-restricted peptides derived from telomerase as previously described^22–25^ (Fig. 1a). We evaluated this response in 170 treatment-naive NSCLC patients and 22 healthy donors (HD) as control. Patients’ main clinical characteristics are depicted in Supplementary Table 1. The presence of spontaneous TERT-reactive CD4^+^ T-cell response was found in 59 NSCLC patients (35%). The median ratio of anti-TERT CD4^+^ T cells was 3.7 (IQR: 2.6–7.5) in responders patients, and the distribution showed two groups with low and high responses (Fig. 1b–d). The frequency of TERT-specific CD4^+^ T cells in age-matched HD was 45% (10/22) in accordance with recent observation reporting high precursor frequencies of tumour antigen-specific CD4^+^ T cells in healthy subjects (Supplementary Fig. 1).^21^ No change of anti-TERT CD4^+^ T-cell response was observed according to patients’ main clinical characteristics, such as age, smoking status, histology and mutational status (Supplementary Table 2). To exclude possible abnormality of antigen-specific memory T cells compartment, we concomitantly measured T-cell reactivity against a mixture of viral peptides (CMV, EBV, Flu) by IFN-γ ELISpot. As expected, the antiviral recall T-cell responses were detected in the majority of patients (81%) and healthy subjects (94%) (Supplementary Fig. 1). Thus, the absence of anti-TERT Th1 response was not related to an intrinsic incapacity of T cells to respond to a stimuli.

Phenotypic characterisation using CCR7 and CD45RA differentiation markers showed that, in contrast to IFN-γ-negative population, TERT-specific CD4^+^ T cells detected by ELISpot assay were CCR7^-^CD45RA^-^ corresponding to effector memory phenotype and also overexpressed activation marker ICOS (Fig. 1e). We also demonstrated CXCR3 but not CCR6 expression on circulating TERT-reactive CD4 T cells, characteristic of a Th1 phenotype (Fig. 1f). Besides the IFN-γ production, these cells concurrently produced TNF-α, and IL-2, but neither IL-4 nor IL-17 in response to TERT stimulation (Fig. 1g). Thus, pre-existing TERT-specific CD4^+^ T cells detected in NSCLC patients are polyfunctional and effector memory Th1 cells.

### The presence of anti-TERT Th1 response is inversely correlated with the level of exhausted PD-1^+^/TIM-3^+^ T cells in NSCLC patients

To identify immune factors likely to influence the circulating anti-TERT Th1 response, we concurrently measured additional blood immune parameters, including lymphocyte subsets, regulatory T cells (T_regs_) and cytokines. As expected, the presence of anti-TERT Th1 response was associated with CD4, but not with CD8, NK or B lymphocyte count in blood (not shown).

**Fig. 2:**
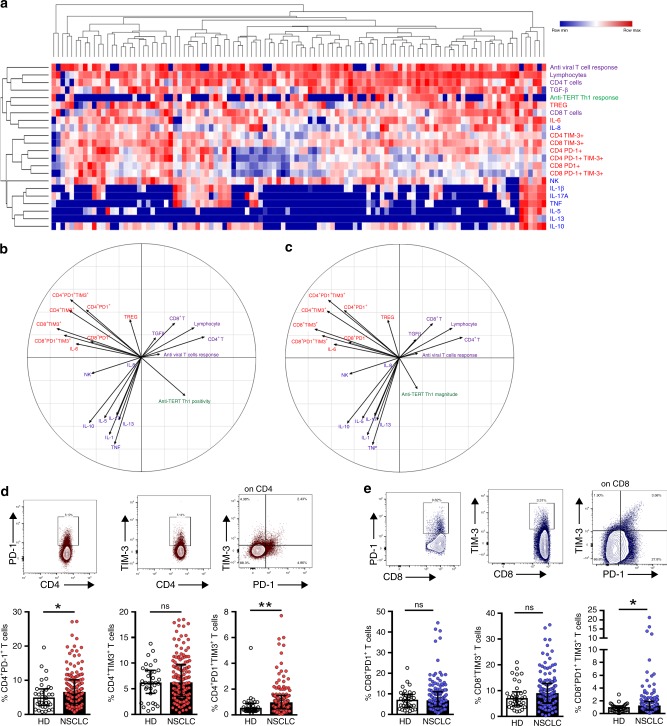
Relationship between TERT-specific CD4^+^ Th1 response and blood immune factors in patients with NSCLC. **a** Heatmap illustrating hierarchical clustering (Euclidean distance) of 22 blood immune parameters (in rows) from NSCLC patients (*n* = 110) (in columns). **b**, **c** Unsupervised principal component analysis (PCA), including frequency (**b**) and magnitude (**c**) of anti-TERT Th1 response in relation to 21 blood immune parameters. **d**, **e** Representative dot plots (top row) show expressions of PD-1 and/or TIM-3 among CD4^+^ T cells (**d**) and CD8^+^ T cells (**e**). Histograms show peripheral T-cell expression levels of PD-1, TIM-3 and PD-1^+^ TIM-3^+^ among CD4^+^ T cells (**d**) and CD8^+^ T cells (**e**) from healthy donors (HD, *n* = 35) and NSCLC patients (*n* = 109). Median and interquartile range (IQR) are indicated (Mann–Whitney test). *P < 0.05; **P < 0.01. ns not significant

Unsupervised clustering analysis revealed that the anti-TERT Th1 response was differently clustered compared with NK cells, inflammatory and inhibitory cytokines, T cells expressing exhaustion markers PD-1 and/or TIM-3 (PD-1^+^/TIM-3^+^), and antiviral T-cell responses. Notably, the heatmap identified a group of patients with low levels of PD-1^+^/TIM-3^+^ T cells, which predominantly included the anti-TERT Th1 responders (Fig. 2a). We also performed PCA to dissect the mutual interactions of these blood immune factors, which revealed that these factors were grouped similarly as in the heatmap clustering (Fig. 2b, c). We identified four distinct immune patterns, which segregated according to the two axes of the PCA. Surprisingly, the circulating anti-TERT Th1 response appeared as an independent factor in the correlation circles, which was opposite to factors that drive immunosuppression, including T_regs_, IL-6 and mainly PD-1^+^/TIM-3^+^ CD4^+^ T cells (Fig. 2b, c).

**Fig. 3:**
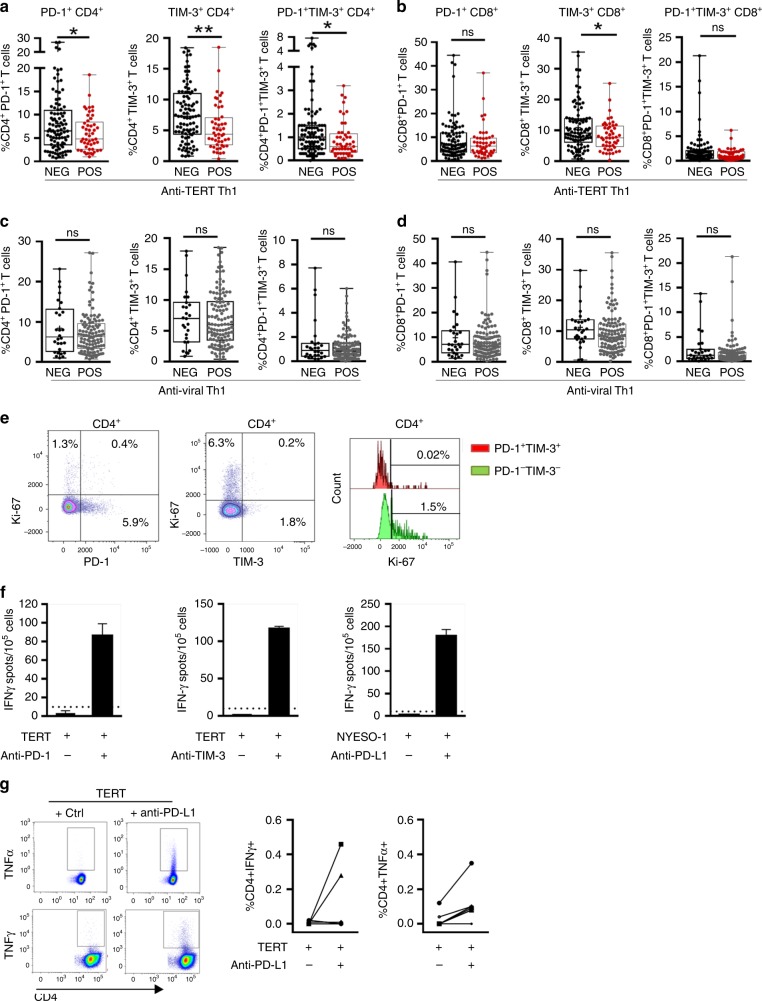
Inverse correlation between the presence of anti-TERT CD4^+^ Th1 response and the level of exhausted PD-1^+^TIM-3^+^ T cells. **a**, **b** Levels of circulating PD-1^+^ and/or TIM-3^+^ CD4^+^ T cells (**a**) and CD8^+^ T cells (**b**) in patients with anti-TERT Th1 response (*n* = 49) and in non-responders (*n* = 96) (Mann–Whitney test). **c**, **d** Levels of circulating PD-1^+^ and/or TIM-3^+^ CD4^+^ T cells (**c**) and CD8^+^ T cells (**d**) in patients with antiviral Th1 response (*n* = 116) and in non-responders (*n* = 28) (Mann–Whitney test). Box spans indicate median and 25th–75th percentile, whiskers indicate the highest/lowest datapoints. **e** Dot plots show Ki-67 staining of unstimulated PBMC from one representative patient. **f**, **g** Blood lymphocytes from patients were stimulated with TERT-derived peptides with or without blocking mAb against PD-L1, PD-1 and/or TIM-3. TERT-specific T cells were measured by ICS or ELISpot. **f** Histograms show IFN-γ spot-forming cells from three representative patients. **g** In left, representative dot plot of TNF-α and IFN-γ-producing CD4^+^ T cells; In right, percentage of IFN-γ and TNF-α-secreting anti-TERT CD4 + T cells (*n* = 6). The data are representative of three independent experiments. *P < 0.05; **P < 0.01. ns not significant

Thus, we focused on the relationship between anti-TERT Th1 response and PD-1^+^/TIM-3^+^ T cells. Firstly, we showed that the rate of both CD4^+^ and CD8^+^ T cells expressing PD-1 or TIM-3 was higher in NSCLC patients than HD. Notably, the circulating T cells co-expressing both PD-1 and TIM-3 (PD-1^+^TIM-3^+^T cells) were preferentially detected at high level in NSCLC patients (Fig. 2d, e). As shown in Fig. 3a, patients with an anti-TERT Th1 response had significantly lower rates of circulating PD-1^+^/TIM-3^+^ CD4^+^ T cells than non-responders NSCLC patients. Particularly, the association with the presence of anti-TERT Th1 response was more pronounced with TIM-3 expressing CD4 and CD8 T cells (Fig. 3b). No obvious relationship was found between the intensity of anti-TERT Th1 response and circulating level of PD-1^+^/TIM-3^+^ CD4^+^ T cells (not shown). In addition, the antiviral T-cell responses were not affected by PD-1^+^/TIM-3^+^ T cells in cancer patients (Fig. 3c, d).

PD-1 and TIM-3 are inhibitory receptors involved in T cells exhaustion.^28,29^ These receptors are commonly expressed on antigen-experienced T cells in the context of a chronic antigen stimulation (virus infection or cancer), and T-cell co-expressing PD-1 and TIM-3 were characterised by a loss of most T-cell functions.^28,29^ So, we investigated the functions of circulating PD-1^+^/TIM-3^+^ T cells detected in NSCLC patients. As expected, we showed low expression of the proliferation marker Ki-67 on PD-1^+^/TIM3^+^ CD4^+^ T cells and in PD-1^+^/TIM3^+^ CD8^+^ T cells (Fig. 3e and not shown). We next evaluated the capacity of these cells to produce Th1-associated cytokines when PD-1 and TIM-3 pathways were disrupted. Hence, the addition of blocking antibodies against the PD-1/PD-L1 axis or against TIM-3 effectively restored IFN-γ and TNF-α production by T cells in response to tumour antigens (Fig. 3f, g). Thus circulating PD-1^+^/TIM-3^+^ T cells dysfunction observed was characteristic of a T-cell exhaustion state.

### NSCLC progression is associated with a decrease of functional anti-TERT Th1 response and accumulation of exhausted PD-1^+^ /TIM-3^+^ CD4^+^ T cells

The concept of cancer immune surveillance suggests that tumour progression is accompanied by an accumulation of immune escape factors, to the detriment of effector T-cell immunity.^1,30^ Therefore, we explored whether NSCLC stage progression was accompanied by changes in the anti-TERT Th1 response and the level of exhausted T cells.

**Fig. 4:**
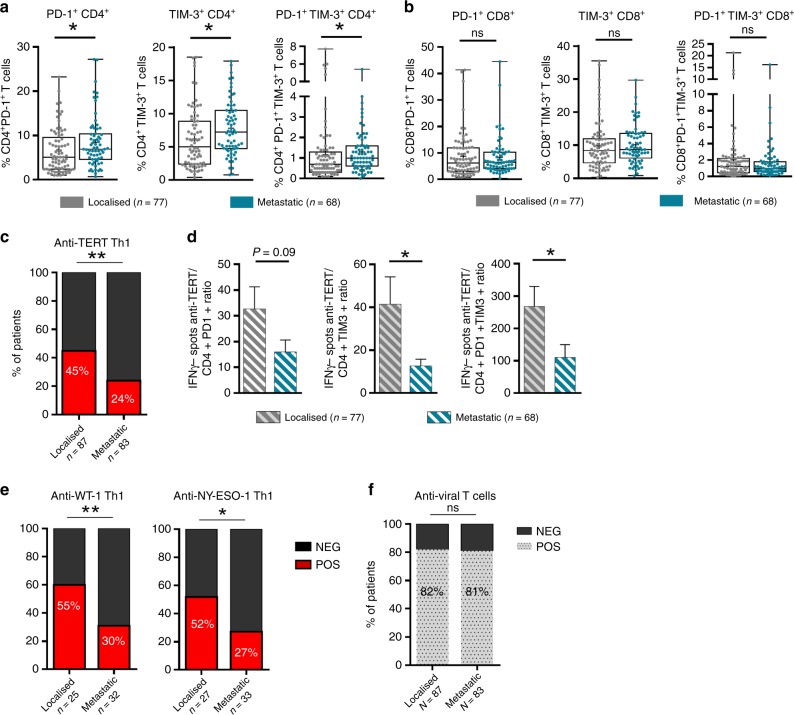
Distribution of circulating exhausted PD-1^+^/TIM-3^+^ T cells and anti-TERT Th1 response across NSCLC stages. **a**, **b** Levels of circulating PD-1^+^ and/or TIM-3^+^ CD4^+^ T cells (**a**) and CD8^+^ T cells (**b**) in localised NSCLC (stages I–III, *n* = 77) and metastatic NSCLC (stage IV, *n* = 68) (Mann–Whitney test). Box span indicates 25th–75th percentiles. Whiskers indicate the highest and lowest datapoints. **c** Frequency of circulating anti-TERT Th1 response in localised versus metastatic NSCLC (χ^2^ test). **d** Ratio of anti-TERT IFN-γ spots to exhausted PD-1^+^/TIM-3^+^ CD4^+^ T cells in localised NSCLC (*n* = 77) and metastatic NSCLC (*n* = 68) (Mann–Whitney test). **e** Frequency of circulating IFN-γ antitumor Th1 response against WT-1, and NYESO-1, in localised versus metastatic NSCLC (χ^2^ test). **f** Frequency of antiviral T-cell responses in localised NSCLC (*n* = 87) versus metastatic NSCLC (*n* = 83) (χ^2^ test). Histograms indicate mean ± SD. *P < 0.05; **P < 0.01. ns not significant

We found a significantly higher rate of exhausted PD-1^+^/TIM-3^+^ CD4^+^ T-cell subsets in metastatic patients (stage IV, *n* = 83) than localised one (stages I–III, *n* = 87). But no obvious stage-related difference was observed with exhausted PD-1^+^/TIM-3^+^ CD8^+^ T cells (Fig. 4a, b; Supplementary Table 3). Conversely, the frequency of anti-TERT Th1 response gradually decreased with stage progression: 44.8% (39/87) versus 24% (20/83) in localised versus metastatic, respectively (*P* *=* 0.004). Accordingly, we found that the ratio of anti-TERT Th1 cells to exhausted PD-1^+^/TIM-3^+^ CD4^+^ T cells was significantly lower in metastatic than localised disease (Fig. 4c, d). The decrease of antitumor T-cell response in the metastatic stage was also found with two additional shared tumour-associated antigens such as WT-1 and NY-ESO-1 (Fig. 4e). In contrast, the antiviral T-cell responses were preserved throughout NSCLC stages, suggesting that these responses were not related to NSCLC evolution (Fig. 4f). Thus, in NSCLC, tumour growth was associated with a decrease of circulating antitumor Th1 responses, but an accumulation of exhausted PD-1^+^/TIM-3^+^ CD4^+^ T cells.

### Anti-TERT Th1 response and exhausted PD-1^+^/TIM-3^+^ CD4^+^ T cells have distinct prognostic value in NSCLC

To assess the prognostic value of circulating anti-TERT Th1 immunity and exhausted PD-1^+^/TIM-3^+^ T cells in this cohort of treatment-naive NSCLC, we considered two groups of patients with low versus high circulating rates of these two immune parameters (see details in the Methods section and Supplementary Table 3).

**Fig. 5:**
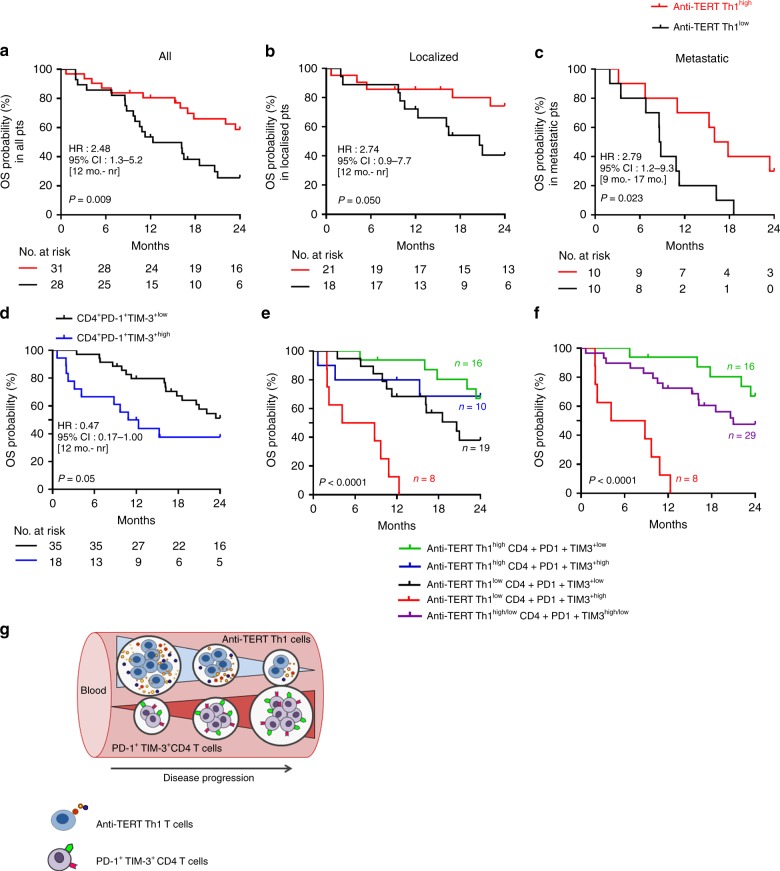
Prognostic value of systemic anti-TERT Th1 response and exhausted PD-1^+^/TIM-3^+^ CD4^+^ T cells in NSCLC. **a**–**c** Association between the level of circulating anti-TERT CD4 Th1 response and overall survival. A threshold (low < 3.7 < high) was defined based on the ratio of TERT-specific IFN-γ spots. Kaplan–Meier curves according to anti-TERT Th1 ratio: in all TERT responders (*n* = 59) (**a**), in localised stages (*n* = 39) (**b**) and in metastatic stages (*n* = 20) (**c**) (log-rank tests). **d** Association between the level of circulating PD-1^+^TIM-3^+^ CD4^+^ T-cell subsets and overall survival. Two groups were determined based on the median rate of exhausted PD-1^+^TIM-3^+^ CD4^+^ T-cell (0.9). Kaplan–Meier curves according to PD-1^+^TIM-3^+^ T cell (log-rank tests). **e**, **f** Patients were classified into distinct groups based on the anti-TERT CD4 Th1 ratio and the median level of PD-1^+^TIM-3^+^CD4^+^T cells. **e**, **f** Kaplan–Meier curves for the following groups: anti-TERT Th1^high^/CD4^+^PD1^+^TIM3^low^ (green), anti-TERT Th1^high^/CD4^+^PD1^+^TIM3^high^ (blue), anti-TERT Th1^low^/CD4^+^PD1^+^TIM3^low^ (black), anti-TERT Th1^low^/CD4^+^PD1^+^TIM3^high^ (red) (log-rank test). Patients in the “blue” and “dark” groups are pooled in (**f**). **g** Schema of the relationship between anti-TERT Th1 immunity- exhausted PD-1^+^TIM-3^+^ CD4^+^ T cells and NSCLC progression

Regardless the immune parameters, the median OS was 13 months in the metastatic population and not reached in localised patients, which is in line with the literature (data not shown).^31,32^ We found that NSCLC patients who exhibited high rates of anti-TERT Th1 cells (anti-TERT Th1^high^) had better median OS compared with patients with anti-TERT Th1^low^ (not reached versus 12 months, *P* *=* 0.009). The two-year survival rate was 2.5-fold higher in the anti-TERT Th1^high^ group compared with the anti-TERT Th1^low^ group (59% versus 22%, respectively; *P* = 0.006) (Fig. 5a). This survival benefit was observed both in localised (not reached versus 21 months, *P* **=** 0.050) and in metastatic NSCLC (median OS of 17 versus 9 months, *P* *=* 0.023) (Fig. 5b, c). Of note, the metastatic patients received first-line therapy with platinum doublet chemotherapy (91%, 74/81) and seven patients with tyrosine kinase inhibitors. None of them have been treated with immune checkpoint inhibitors. So the difference in survival in this population could not be associated with the differences in first-line treatment (not shown).

Next, the prognostic value of exhausted PD-1^+^/TIM-3^+^ T cells was evaluated. No association was found with PD-1^+^/TIM-3^+^ CD8^+^ T cells and clinical outcome in this cohort (not shown). Among immune responder patients, the presence of high rate of exhausted PD-1^+^TIM-3^+^ CD4^+^ T cells was associated with a poor survival, the median OS was 12 months in this group and not reached in CD4^+^PD-1^+^TIM-3^+low^ group (*P* = 0.05). This effect was particularly observed in patients with localised disease (Fig. 5d; Supplementary Fig. 2). Although, the rate of T cells expressing the single receptor PD-1 did not affected patients clinical outcome, some trend of a negative association was found with high level of TIM-3^+^ CD4^+^ T cells (Supplementary Fig. 2). Univariate and multivariate Cox analysis confirmed that both the circulating anti-TERT Th1 cells and PD-1^+^TIM-3^+^ CD4^+^ T cells were significantly but inversely associated with the survival and independent of metastatic stage (Table 1).

**Table 1:** Cox proportional analysis for overall survival

Cox regression analyses		Univariate			Multivariate		
*n* = 59	*N*	HR^a^	95% Cl^b^	*P-*value	HR^a^	95% Cl^b^	*P-*value
*Anti-TERT Th1 response*							
Low (ratio < 3.7)	31	1			1		
High (ratio > 3.7)	28	0.396	0.192–0.817	0.0121	0.206	0.083–0.511	0.0007
*Stage*							
Localised (I–III)	39	1			1		
Metastatic (IV)	20	3.245	1.605–6.558	0.0010	3.545	1.579–7.960	0.0022
*Histologic subtype*							
Adenocarcinoma	30	1					
Squamous cell carcinoma	12	0.902	0.396–2.054	0.8056			
*PD-1* ^*+*^ *CD4 T cells*							
Low	32	1					
High	21	0.973	0.445–2.128	0.9462			
*TIM-3* ^*+*^ *CD4 T cells*							
Low	36	1					
High	17	1.880	0.872–4.057	0.1075			
*PD-1+/TIM-3* ^*+*^ *CD4 T cells*							
Low	35	1			1		
High	18	2.126	0.980–4.609	0.0562	2.793	1.173–6.649	0.0203
*PD-1* ^*+*^ *CD8 T cells*							
Low	28	1					
High	25	0.977	0.457–2.089	0.9523			
*TIM-3* ^*+*^ *CD8 T cells*							
Low	33	1					
High	20	0.660	0.288–1.510	0.3253			
*PD-1+/TIM-3* ^*+*^ *CD8 T cells*							
Low	31	1					
High	22	1.457	0.681–3.119	0.3323			

Univariate and multivariate analysis for OS based on anti-TERT Th1 response, exhausted PD1^+^TIM-3^+^ T cells and main clinical characteristics

^a^Hazard ratio

^b^Confidence intervals

Based on our findings, we stratified patients into three prognostic groups (best, intermediate and poor) according to these two immune parameters. The best group represents patients with anti-TERT Th1^high^/exhausted CD4^+^PD-1^+^TIM-3^+low^ profile (median OS not reached), the group of patients with anti-TERT Th1^low^/exhausted CD4^+^PD-1^+^TIM-3^+high^ profile had a poor prognosis (median OS = 4 months) and the third group with intermediate survival had a similar evolution of the two immune parameters (high/high or low/low) (Fig. 5e, f). This stratification highlighted that anti-TERT Th1 response plays a strong antitumor protective role over the level of exhausted PD-1^+^/TIM-3^+^ T cells. Collectively, ours results indicated that the level of anti-TERT Th1 response and exhausted PD-1^+^TIM3^+^CD4^+^ T cells have distinct prognostic value in NSCLC, so that the decrease of functional anti-TERT Th1 cells and increase of exhausted PD-1^+^TIM-3^+^CD4^+^ T cells were associated with disease progression (Fig. 5g).

## Discussion

Numerous aspects of CD4^+^ T-cell biology suggest that these cells are required for effective antitumor immunity and immunotherapy. Importantly, they have the ability to eliminate cancer cells, mainly in an indirect manner by influencing the TME.^2,5^ Despite these critical antitumor immune functions, the clinical significance of CD4^+^Th1 cells remains scarcely exploited. In this study, we performed a comprehensive systematic analysis of circulating antitumor CD4^+^ Th1 response across NSCLC clinical stages, using anti-telomerase CD4^+^ T-cell response as a surrogate marker of antitumor Th1 immunity. This approach enables dynamic monitoring of IFN-γ-producing tumour-reactive CD4^+^ T cells using an ELISpot assay, regardless of HLA type.^22–26^ Around 35% of therapy-naive NSCLC patients harboured circulating anti-TERT Th1. This proportion was in line with our previous findings, and with the overall prevalence of tumour-infiltrative lymphocyte (TIL) positivity identified in many human cancers.^14^ The frequency of patients with circulating anti-TERT Th1 immunity decreased with NSCLC progression, being only 24% among metastatic stage IV compared with 45% and 55% in localised I–III and I–II stages, respectively. This lower frequency found in metastatic patients compared with localised ones and in healthy subjects (45%) suggested that tumour progression is associated with a defect of pre-existing anti-TERT CD4^+^ Th1 immunity. Indeed, a similar decrease of T-cell responses directed against NY-ESO1 and WT-1 was observed, in metastatic stage. Interestingly, we demonstrated that the presence of a strong pre-existing anti-TERT Th1 response in blood was significantly associated with better OS in NSCLC patients with both localised and metastatic disease. In line with our findings, previous studies have also reported clinical benefits of pre-existing or therapy-induced circulating IFN-γ-secreting CD4^+^ T cells against NY-ESO-1, 5T4, SOX2 and HPV-16.^15,33–36^ These data support that a pre-existing antitumor CD4^+^ Th1 signature in peripheral blood may confer tumour protection. However, given the descriptive nature of our study, it would be needed to validate these findings in another external cohort. Nevertheless, these results prompt us to stimulate anti-TERT CD4^+^ T-cell response in NSCLC patients (NCT2818426).

The protective role associated with anti-TERT Th1 response could be explained by the functional characteristics of these cells. Indeed, TERT-reactive CD4^+^ T cells detected in NSCLC patients were effector memory cells and polyfunctional Th1-polarised capable to produce effector cytokines such as IFN-γ, TNF-α and IL-2. Indeed, many evidences support that CD4^+^ Th1 subset orchestrates cell-mediated immunity against cancer cells mainly by enhance tumour-specific CD8^+^ T-cell functions, survival and migration in the TME. These antitumor roles of CD4^+^ Th1 cells are mainly driven by the triad of cytokines, such as IFN-γ, TNF-α and IL-2.^5,6^ Furthermore, a clinical benefit associated with Th1-polarised signature in the TME has been reported in many human cancers.^13^

Prognostic value associated with CD8^+^ TIL represents the current dogma of adaptive immune signature against cancers.^13,14,37^ Here, circulating CD8^+^ T-cell responses against HLA-A*0201 and HLA-B7-restricted peptides from telomerase were detected in 13% (8/60) of patients using the ELISpot assay (not shown). This low frequency, compared with telomerase-specific CD4^+^ Th1 response, could be related to the use of highly selected and promiscuous HLA class II-binding peptides that cover more than 85% of the population,^22,24^ or to the preferential localisation of these effector cells in the tumour. Since Th1-polarised CD4^+^ T cells reportedly control CD8^+^ T cells migration in the TME,^6,8^ it could be expected that patients with potent circulating anti-TERT Th1 response showed high CD8^+^ TIL, but this hypothesis deserves future investigations.

Targeting tumour-reactive CD4^+^ T cells in peripheral blood raises questions regarding the choice of tumour-associated antigens.^20^ Telomerase activity maintains tumour cell immortality by protecting cancer cells from telomere-dependent cell death, and telomerase overexpression is reported in the majority of human cancers.^38,39^ The critical activities of telomerase during the oncogenesis process may also prevent immune escape via an antigen-loss mechanism.^1^ Thus, the good prognostic value of a CD4^+^ T-cell response against telomerase highlights the critical functions of this antigen.^40^

To search for factors influencing the presence of a TERT-reactive CD4^+^ Th1 response in peripheral blood, we found an inverse correlation between the presence of anti-TERT Th1 response and the level of exhausted PD-1^+^/TIM-3^+^ T cells, particularly T cells expressing TIM-3. The co-inhibitory receptors PD-1 and TIM-3 are well-known markers of T-cell exhaustion, which are expressed on tumour antigen-experienced T cells.^28,41^ Previous reports in lung cancer, showed co-expression of PD-1 and TIM-3 on TIL are associated with poor prognosis.^42,43^ Similar observations are reported in several other cancers, underlining the negative impact of exhausted T cells in cancer immunosurveillance.^44,45^ This state of exhaustion is characterised by the loss of most T-cell functions, including effector cytokines production, proliferation and cytotoxicity^28^, and likely explains the poor prognosis associated with exhausted T-cell accumulation. The apparent anti-exhaustion role of the anti-TERT Th1 response is in agreement with findings in mice, showing that CD4^+^ helper T cells protect CD8^+^ T cells from exhaustion by downregulating expressions of the co-inhibitory receptors PD-1, TIM-3 and Lag-3.^8^ In a recent investigation using deep single-cell RNA sequencing to analyse the T-cell landscape in lung adenocarcinoma, the authors found that a high ratio of pre-exhausted to exhausted PD-1^+^TIM-3^+^ T cells was associated with better prognosis. They also reported that the TILs from lung adenocarcinoma had a gene signature similar to that of blood T lymphocytes.^43^ These observations, together with our findings, indicate that circulating tumour-reactive T cells may reflect occurrences in the tumour.^30,34,46^

Many reports emphasise the need for predictive biomarkers, in the hope of increasing responses to immune checkpoint inhibitors.^47,48^ Current biomarkers are focused on PD-L1 expression, CD8^+^ TILs and tumour mutational burden, however, emerging blood-based biomarkers have attracted considerable interest.^49,50^ Indeed, liquid biopsy offers numerous advantages, including the potential for serial assessment and dynamic monitoring with minimal invasiveness. Accordingly, recent studies in NSCLC and melanoma reported pharmacodynamic changes of circulating Ki-67^+^PD-1^+^CD8^+^ T cells following anti-PD-1 therapies.^46,51,52^ Another report also demonstrated that high circulating central memory T cell to effector T-cell ratios were associated with better clinical outcome in NSCLC receiving anti-PD-1 therapy.^53^ Our previous findings also support the critical role of treatment induced anti-TERT CD4^+^ Th1 immunity. For example, reactivation of a systemic anti-TERT Th1 response after everolimus treatment improved patient survival in renal cell carcinoma by counterbalancing immune-suppressive T_regs_.^25^ More recently, the induction of anti-TERT Th1 response after immunogenic polychemotherapy significantly increased progression-free survival in patients with anal squamous cell carcinoma.^26^ Thus, we strongly believed that the presence of a functional anti-TERT CD4^+^ Th1 response may create a suitable inflamed environment for immune checkpoint inhibitor actions, in turn, improving therapy efficacy. The monitoring of anti-TERT Th1 response as potential biomarker for immunotherapy is currently evaluated in several cancers (NCT02840058).

In conclusion, our present study provides a new blood-based tool for NSCLC patients stratification. This approach could be rapidly deployed in routine clinical practice, and is likely also applicable to other cancers.

## Supplementary information

Supplemental Materials and methods

## Data Availability

The data sets generated during and/or analysed during this study are available from the corresponding author on reasonable request.
