# Supplementary material for: Distinct prognostic value of circulating anti-telomerase CD4+ Th1 immunity and exhausted PD-1+/TIM-3+ T cells in lung cancer
Source: Br J Cancer. 2019 Jul 30;121(5):405–16. doi: 10.1038/s41416-019-0531-5 (PMC6738094; doi:10.1038/s41416-019-0531-5)
Supplement: Supplementary file 1 — Supplemental Materials and methods [file 41416_2019_531_MOESM1_ESM.docx]

**Supplementary Materials and Methods**

List of monoclonal antibodies used for flow cytometry:

**Supplementary Table 1**

**Main clinical characteristics of NSCLC patients**

**Supplementary Table 2**

**Circulating anti-TERT Th1 responses according to patients’ main clinical characteristics.**

**Supplementary Table 3**

**Level of circulating exhausted PD-1^+^TIM-3^+^T cells in NSCLC patients**

*P*, Mann & Whitney test (Localized versus Metastatic)

**Supplementary Fig.1**

**Supplementary Figure 1**: T cells responses against TERT and virus in age-matched healthy donors and NSCLC patients. **a**, Frequency of anti-TERT and anti-viral T cell responses in 22 age-matched healthy donors. **b**, Frequency of anti-TERT and anti-viral T cell responses in 170 NSCLC patients.

**Supplementary Fig.2**

**Supplementary Figure 2:** Association between the levels of circulating exhausted PD1^+^ and/or TIM-3^+^ CD4^+^ T-cell subsets and overall survival. Two groups were determined based on the median rate of PD-1^+^ or TIM-3^+^ CD4^+^ T cell subset (see Supplementary Table 3). **a and b**, Kaplan–Meier curves according to the median rate of PD-1^+^TIM-3^+^ CD4 T cells: in localized stages (*n* = 35) (**a**), and in metastatic stage (*n* = 18) (**b**) (log-rank tests). **c and d**, Kaplan–Meier curves according to the level of PD-1^+^ CD4^+^ T-cell (c) or TIM-3^+^ CD4^+^ T-cell (d) (log-rank tests).
